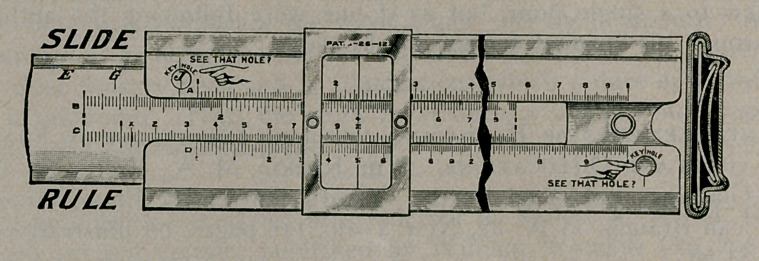# Book Reviews

**Published:** 1914-02

**Authors:** 


### BOOK REVIEWS.

*Books mentioned may be inspected at and ordered through this office. So far as possible, books received in any month will be reviewed in the issue of the second month following.*

Publishers will please mark the price of each book on the front fly leaf.

Essentials of Nervous Diseases and Insanity. John C. Shaw, M. D. Late Clinical Professor of Diseases of the Mind and Nervous System, Long Island College Hospital Medical School. Fifth revised edition by Louis Casamajor, M. A. M. D., New York. 187 pages, illustrated.

Essentials of Gynaecology. Edwin B. Cragin, M. D., Professor of Obstetrics and Gynaecology, College of Physicians and Surgeons, New York. Eighth revised edition by Frank S. Matthews, M. D., New York, 240 pages, illustrated.

Essentials of Bacteriology. M. V. Ball, M. D., Warren, Pa., assisted by Paul G. Weston, M. D., Warren, Pa. Seventh revised edition, 321 pages, 118 illustrations.

All of the above are published by the W. B. Saunders Co. of Philadelphia and London, at $1.00 per volume, being part of the blue covered series of Saunders' Question Compends. The number of revisions attests both the popularity of the works and the fact that the series is being kept fully in accordance with modern discoveries. It is unnecessary to emphasize the necessity of compends of this nature for students not to state that the stock criticism of quiz work, in class or by text books, should be directed toward general methods of medical education and not toward means that have developed in accordance with such methods. But it may not be out of place to call attention to the great value of properly prepared books of this sort, to refresh the memory of the practicing physician and to enable him to place in their proper places, the details which he is constantly absorbing from periodic literature. The text books of this series are well adapted to both these functions. Many of our readers will feel personal interest in the last named book, since Dr. Ball was for some time in practice in Buffalo and an officer of one of the societies that has become a section of the Buffalo Academy of Medicine.

THE NARCOTIC DRUG DISEASES AND ALLIED AILMENTS. George E. Pettey, M. D., Memphis. Published by the F. A. Davis Co., Philadelphia. 516 pages.

Interesting points in this work are the tolerance for drugs established, the dosage of antagonistic drugs required (e. g., ¼ grain of strychnine, repeated up to a grain in a day in morphine cases) the sceptic views as to specific formulæ, the broadness of scope necessary in successful treatment, including diet, baths, cathartics, psychic suggestion, etc. The writer has not attempted to solve any of the problems of narcotic addiction in an easy manner nor has he attempted to be original at the expense of ignoring the valuable results of accumulated experience. He has, therefore, consulted the ultimate welfare of the patient and of his attendant, not the convenience of the reviewer nor of the man who is looking for some easy and brief way out of difficulties. It is a painstaking book for a painstaking reader.

---

TRANSACTIONS OF THE AMERICAN SURGICAL ASSOCIATION. Volume 31, for 1913, printed under the editorship of Dr. Archibald MacLaren, Recorder, St. Paul, Minn., by Wm. J. Dornan, Philadelphia. 622 pages, illustrated.

The book contains lists of officers and members, of members deceased during the year and a report of the committee on fractures, beside the monographs presented by members at the meeting in Washington, May 1913. It would be invidious to select a few of these for review, impossible to do justice to all. We can only say, therefore, that the papers are of the highest order and that both the editor and the printer have discharged their duties well.

---

CUNNINGHAM'S TEXT BOOK OF ANATOMY, 4th edition, enlarged and re-written, edited by Arthur Robinson, M. D., F. R. C. S. of Edinburgh. 1596 pages, 1124 illustrations, 637 in colors, and 2 plates. William Wood & Co., New York, $6.50 cloth, $7.50 half morocco.

Owing to the death of Drs. Cunningham, Birmingham and Young, a new editorship and the selection of new authors for various sections, have been necessary. The detailed credit is given in the preface. While anatomy appears on superficial consideration, to be a fixed branch of medical science, marked advances have been made in our understanding of embryology, of the ductless glands and of many other structures, at least in detail. Thus it can no longer represent the investigation or even the collation of any one man, nor can it be satisfactorily presented by a mere reprinting of former editions nor even by supplementing the text with illustrations of higher technic. The Basle anatomic terminology has been used throughout except in a few instances in which terms have been shown to be incorrect under older interpretations. Every effort has been made, not only to incorporate the most exact and most modern researches as to structure but to facilitate the work of the student by clear literary style and by illustrations which reduce eye strain to a minimum. Thus the work is, at once, a monumental treatise, and a practical compend.

---

Text Book of Histology by Frederick R. Bailey, A. M., M. D., 4th revised edition, published by William Wood & Co., New York, 644 pages, 384 illustrations, $3.50.

The first 38 pages deal with histologic technic, a short section on the cell follows and then longer sections on the tissues and the organs. The text is lucidly written and the cuts distinct while the fact that the book has passed through three previous editions, attests its popularity and genuine merit.

---

The Practitioner's Practical Prescriber and Epitome of Symptomatic Treatment, D. M. MacDonald, M. D., Medical Officer of Health, Leven, Fife. Oxford University Press, American Branch, 35 W. 32, N. Y. 198 pages, $1·50.

About two-thirds of this little book is arranged alphabetically, by diseases, with brief indications for treatment, the prescriptions being, of course, in the apothecary's system and alluding to the B. P. The remainder of the book consists of posologic tables, discussion of incompatibility, etc., diet tables, list of diseases treated by X-ray, a form for post mortem examination reports and other miscellany.

---

Practical Prescribing with Clinical Notes. Arthur H. Pritchard, M. R· C. S., L. R. C. N., R. N., retired. Oxford University Press, American Branch, 35 W. 32, N. Y. 307 pages, $2.

The diseases covered are grouped as constitutional, infective and of various organs. The clinical method, with description of case, prescription sheet and discussion of treatment and symptoms is mainly followed. Interesting notes of experience with certain drugs and methods of treatment, are interpolated.

In many parts of the book, the two-column arrangement of prescription sheet on one side and course of the disease on the other is employed. The arrangement of the work seems unique and valuable as affording the reader the benefit of actual experience with cases, not in the ordinary American form of clinics, which except for history and notes of progress limit the view to a single hour, but as if one were following the author through his hospital service day by day.

---

The Elements of Bandaging and the Treatment of Fractures and Dislocations. Wm. Rankin, M. A., M. B., Ch. B., Glasgow; published by the Oxford University Press, American Branch, 35 W. 32, New York; 116 pages, 68 illustrations; $1.50.

This is a brief treatise, divided into parts as indicated in the title, but 16 pages being given to Dislocations. The part on Fractures is headed with an Armamentarium of appliances necessary. The general principles of the subject are well presented and the technicalities of particular fractures, etc., are discussed much more completely than the size of the book leads one to expect.

---

Poisons and Habit Forming Drugs. Martin I. Wilbert and Murray Galt Motter, Technical Assistants, Hygienic Laboratory; Bulletin No. 146, U. S. Public Health Service.

This is a digest of laws and regulations relating to the possession, use, sale and manufacture of poisons and habit forming drugs, enacted during 1912 and 1913 and now in force in the U. S.

---

E. Merck's Annual Report of Recent Advances in Pharmaceutical Chemistry and Therapeutics. Vol. 26, 1912; published by E. Merck, Darmstadt.

This should not be mistaken for an ordinary price list or advertisement. It is an elaborate collation of highly scientific work along various lines. The first 71 pages are devoted to lecithin, the bibliography occupying 20 pages. About 400 pages are then given to an alphabetic consideration of new drugs and of new methods and uses of old drugs. The indexes and supplement occupy about 70 pages. The work is in English. The Report will be sent on receipt of 15 cents to pay carrying charges.

The Slide Rule Simplified, by George W. Richardson, Consulting Engineer and Manufacturer of Slide Rules, 4214 24th Place, Chicago. The book, of 52 pages, paper covers, $1.00; flexible silk binding, $0.50; the rule, $2·50, in card board case; leather case, $0.50.

Aside from professional engineers, most men forget the advantages and practical uses of higher mathematics and very few have even heard of the slide rule. The general principle of this instrument is that by the comparison of sliding scales, divided by lines, various problems involving multiplication, division, raising to powers and extracting roots, determination of sines, cosines, logarithms, etc., may be solved approximately, with considerable rapidity. Most such instruments are expensive and complicated. The present instrument is quite inexpensive and comparatively simple, though it is proper to warn the one unfamiliar with it, that no slide rule can be made that is as simple as a calendar or a chart for diagnosing bacteria. Some of the uses to which the physician may put this rule are as follows: calculation of simple and compound interest, (which will save considerable sums in checking the off-hand statements as to the value of life insurance as an investment) conversion of metric into apothecary units and vice versa, calculations involving titration and molecular weights, estimation of food values, calculation of gasolene in horizontal cylindric tanks, the depth of gasolene being the co-versed sine of the arc filled, electric calculations of various kinds. Very little has been done as to the mathematics of physiology and other medical sciences and we trust that some mathematician will make a special study of medical quantitation. We have been impressed with the marked practicability of dosage of alkalies, based on titrations of acid secretions and excretions, notably stomach contents and urine and while it is unlikely that quantitation can ever be absolutely accurate for problems involving a vital factor, it is altogether probable that they are susceptible of much greater accuracy than is usually believed.

Progressive Medicine, edited by H. A. Hare and L. F. Appleman; published by Lea & Febiger, Philadelphia and New York; quarterly; $6 per annum.

The present volume deals with the current literature on the Digestive Organs, Kidneys, Peritoneum, Genito-urinary Organs, Surgery of the Extremities, Shock, Anaesthesia, Infections, etc., and concludes with a Practical Therapeutic Referendum. The contributors are Joseph C. Bloodgood, Charles W. Bonney, John R. Bradford, Edward H. Goodman and H. R. M. Landis. Owing to the wide range of subjects covered and the fact that the work is, in itself, a review, we select no special subjects for mention but endorse in the highest terms the quality of the work as a whole.

---

Practice of Medicine. James Tyson, M. D., LL. D., and M. Howard Fussell, M. D., of Philadelphia. P. Blakiston's Son & Co., Philadelphia; 1211 pages, 6 plates and 179 other illustrations.

An earlier edition of this work has been on our shelves for years, and on the whole, we have come to regard it as the easiest for reference, the one most likely to furnish definite information as to the habitat, semelincidence or the reverse of disease, problems sub judice, etc., and in short the one best adapted for "brushing up" on half forgotten points. While it may be that we are prejudiced by the natural and proper regard for a favorite teacher, we are inclined to believe that the practicability of Dr. Tyson's work has been due to three factors: his long experience as a teacher of pathology at a period when specialization in this branch did not exclude attention to practice; the fact that his teaching of the practice of medicine was mainly as a clinical and not a didactic professor; his official service as Dean and kindly disposition, which brought him more into contact with medical students and young medical practitioners than the average teacher of medicine, and thus enabling him to comprehend to the fullest, the methods most successful in imparting technical knowledge. The fact that the work has reached its sixth edition is ample proof of its value. Dr. Tyson has fully realized and cordially conceded the advantages of bringing to the task of revision, the services of younger blood, as well as of securing expert assistance in certain chapters.

Surgery of the Upper Abdomen. John B. Deaver, M. D., Sc. D., LL. D., and Astley Paston Cooper Ashhurst, A. B., M. D·, Vol. 2, Surgery of the Gall Bladder, Liver, Pancreas and Spleen; 499 pages, 52 illustrations, including colored plates, $5; published by P. Blakiston's Sons & Co.

At the risk of continuing too far, the personal reminiscences suggested by the previous review, we may say that we enjoyed the clinical teaching in surgery of Dr. Deaver as well as of the late John Ashhurst Jr. No experiment has more amply justified a departure from precedent than the appointment of Dr. Deaver to the faculty of the University of Pennsylvania, although, at the time, the custom had been to select the faculty from a very limited area in municipal geography.

This book would appeal favorably to the reviewer if it came from a previously unknown source. It is more than a surgery and is of value to the medical practitioner, perhaps to a greater degree than to the surgeon. The latter, trained in technic, having the case in hand essentially for operation, and compelled to follow emergent indications and to meet mechanic factors with personal ingenuity, may feel that he needs this guide less than an increase of personal experience although both in anticipation and in retrospect, it will afford him wise counsel. But, under existing conditions, the responsibility for visceral surgery and especially of this region, rests largely on the internist. He it is who needs most to know what conditions are likely to be present, in a more definite sense than can be determined by external and physiologic methods, and what can be expected from surgical intervention. The authors, while dwelling sufficiently on surgical detail, and justly deriving support from their previously acquired authority, have refrained from limiting themselves to operative technic and to personal experience, much less to generalities. Elaborate tables are presented, as to the bacteriology of series of cases, exact location of calculi, results of different methods of treatment and numerous other points on which the medical practitioner must weigh the indications for surgery; and each section is followed by a bibllography. Thus, practically every moot point is decided by actual evidence.

---

Diagnostic Wall Chart—Sputum. Published by the Palisade Mfg. C., Yonkers, N. Y· Typic microscopic fields are displayed in a circle. By pointing a movable arrow at any one, a description of the field is uncovered by a disc attached to the arrow. Any physician interested will receive a chart on application to the company.

We have noticed an unfavorable comment in one of our contemporaries, which takes the ground that the fields depicted will not correspond to actual findings and that an attempt to make diagnoses will lead to error. In the literal sense, there is probably some truth in this but the same criticism applies to illustrations in the most approved text books. Typic findings are rare. If actual microscopic appearances are reproduced, they cannot apply exactly to other cases and they confuse the mental picture more than if theoretic types are employed. In order that the mental picture be clear, it is necessary not only to separate into its components the very mixed picture obtainable in the great majority of cases but to accentuate somewhat, the features that must be noted by the eye. Probably no one would have less expectation of suggesting this chart for the guidance of expert pathologists than the publishers who would, equally probably, be the last to imagine that the non-expert would pose as an expert microscopic diagnostician, simply on the basis of the possession of the chart. But, they have presented in convenient form, a resume of the principal structural findings to be looked for in the sputum. It gives a good general conception of the subject to the man who does not expect to use the microscope for fine diagnostic purposes and it will clarify the mental picture and refresh the memory of the man who uses such methods with average skill but who makes no pretensions to expertness.

---

PRINCIPLES OF MICROBIOLOGY, A Treatise on Bacteria, Fungi and Protozoa Pathogenic for Domesticated Animals. Veranus Alva Moore, B. S., M. D., V. M. D., Ithaca; published by Carpenter & Co., Ithaca; 506 pages, 101 illustrations; $3.50.

Great attention is paid to general technic, methods of classification, preparation of media, use of animal inoculation, etc., which occupy about a third of the book. An ingenious designation of bacteria modeled after the decimal system of library cataloguing is suggested, extending from hundreds to six decimal places, each figure having a special significance, hundreds applying to spores, tens to aerobic and anaerobic growth, units to action on gelatin, tenths to action on dextrose, etc. The botanic classification of bacteria as if bacillus, streptococcus, etc., indicate a genus is, of course, purely tentative. The allusions to filterable viruses, especially the observations that certain of these are not filterable through perfect filters, are interesting. The detailed description of bacteria and other microorganisms are exact and lucid and, dealing with the domesticated animals, present many points with which the medical profession is unfamiliar, which throw light on general principles of microbiology and suggest that a broader view of the science might discover general laws where we now depend upon scattered observations.

Without regard to the particular subject taught, the author shows didactic skill of the highest order and it is in order to acknowledge the value of contributions from veterinary medicine to general medical science. The publishers have also done their part well.

---

TRANSACTIONS OF THE AMERICAN GASTRO-ENTEROLOGICAL ASSOCIATION. Sixteenth Annual Session, Washington, May 5 and 6, 1913. Fifteen monographs are contained in this volume, covering various phases of the subject, there being a commendable tendency to consider outlying and unsolved problems rather than those making up the majority of practice of the members.

---

THE ADVERTISER'S HANDY GUIDE, 1913, Compiled and published by the Morse International Agency, 449 Fourth Ave, N. Y.; 810 pages; $2.00.

This contains lists of newspapers by states and provinces, of medical, religious, agricultural and other publications by classes, with statistics of circulation, etc. We are, naturally especialy interested in the medical list and note the shrinkage of one of our contemporaries from 7,000 to less than 1,600, also, the remarkably large circulation of some essentially local publications in areas of sparse population. There is a surprising lack of relation between journalistic excellence or even a meeting of a popular demand, and circulation. Generally speaking, the organization journals, have a large proportionate circulation, being sent to every member, whereas, owing to subscription sharing, independent journals cover, on the average about two physicians to a copy. We have made a substantial gain in circulation since furnishing statistics. The Morse Agency are to be complimented on having prepared a neat and convenient guide for those interested in publicity.

---

MORSE'S CASE HISTORIES IN PAEDIATRICS, published by W. M. Leonard, Boston, revised in January issue, page 377. The price was stated as $3.00. It should have been $5.50.

Genito-Urinary Diseases and Syphilis—By Edgar G. Ballenger, M. D., Adjunct Clinical Professor of Genito-Urinary Diseases, Atlanta Medical College; Editor Journal-Record of Medicine; Urologist to Westley Memorial Hospital; Genito-Urinary Surgeon to Davis-Fisher Sanatorium; Urologist to Hospital for Nervous Diseases, etc., Atlanta, Ga., assisted by Omar F. Elder, M. D. The Wassermann Reaction by Edgar Paullin, M. D. Second edition revised; 527 pages with 109 illustrations and 5 colored plates; price $5.00 net; E. W· Allen & Co., Atlanta, Ga.

The author speaks somewhat guardedly of the specificity of the Ducrey-Unna streptobacillus. The explanation and qualification of Colle's law is well put, though we think that one not already familiar with it, would not quite understand the statement that Profeta's law is the converse of Colles's. The Wassermann reaction and various modifications are discussed at length and with proper qualifications though a plus plus reaction is considered practically pathognomonic. Plus minus reactions may occur in normal individuals. Extra-genital and non-venereal lesions are well discussed although for gonorrhoea, the general statement of their rarity is all that we find. The book is thoroughly practical and complete without being replete with unnecessary details.

We express our appreciation of the blank review slip enclosed, bearing the usual statements at the top and leaving space below for review, even an extra sheet being folded over for the carbon copy. We trust that other publishers will follow this convenient method.

## Figures and Tables

**Figure f1:**